# Antibacterial Activity against *Staphylococcus Aureus* of Titanium Surfaces Coated with Graphene Nanoplatelets to Prevent Peri-Implant Diseases. An In-Vitro Pilot Study

**DOI:** 10.3390/ijerph17051568

**Published:** 2020-02-29

**Authors:** Nicola Pranno, Gerardo La Monaca, Antonella Polimeni, Maria Sabrina Sarto, Daniela Uccelletti, Erika Bruni, Maria Paola Cristalli, Domenico Cavallini, Iole Vozza

**Affiliations:** 1 Department of Oral and Maxillo-Facial Sciences, Sapienza University of Rome, 00100 Rome, Italy; nicola.pranno@uniroma1.it (N.P.); antonella.polimeni@uniroma1.it (A.P.); iole.vozza@uniroma1.it (I.V.); 2 SNN Lab, Sapienza Nanotechnology & Nano-Science Laboratory, Sapienza, University of Rome, 00100 Rome, Italy; mariasabrina.sarto@uniroma1.it (M.S.S.); domenico.cavallini@uniroma1.it (D.C.); 3 DIAEE, Department of Astronautical, Electrical, Energy Engineering, Sapienza University of Rome, 00100 Rome, Italy; 4 BBCD, Department of Biology and Biotechnology, Sapienza University of Rome, 00100 Rome, Italy; daniela.uccelletti@uniroma1.it (D.U.); erikabruni89@gmail.com (E.B.); 5 Department of Biotechnologies and Medico Surgical Sciences, Sapienza University of Rome, 00100 Rome, Italy; mariapaola.cristalli@uniroma1.it

**Keywords:** colloidal suspension, dental implant, *Staphilococcus aureus*, graphene nanoplatelets antibacterial surface

## Abstract

Dental implants are one of the most commonly used ways to replace missing teeth. Nevertheless, the close contact with hard and soft oral tissues expose these devices to infectious peri-implant diseases. To prevent such infection, several surface treatments have been developed in the last few years to improve the antimicrobial properties of titanium dental implants. In this in-vitro pilot study, the antimicrobial activity of titanium surfaces coated with different types of graphene nanoplatelets are investigated. Six different colloidal suspensions of graphene nanoplatelets (GNPs) were produced from graphite intercalated compounds, setting the temperature and duration of the thermal shock and varying the number of the exfoliation cycles. Titanium disks with sand-blasted and acid-etched surfaces were sprayed with 2 mL of colloidal GNPs suspensions. The size of the GNPs and the percentage of titanium disk surfaces coated by GNPs were evaluated through a field emission-scanning electron microscope. The antibacterial activity of the specimens against *Staphylococcus aureus* was estimated using a crystal violet assay. The dimension of GNPs decreased progressively after each sonication cycle. The two best mean percentages of titanium disk surfaces coated by GNPs were GNPs_1050°/2_ and GNPs_1150°/2_. The reduction of biofilm development was 14.4% in GNPs_1150°/2_, 20.1% in GNPs_1150°/3_, 30.3% in GNPs_1050°/3_, and 39.2% in GNPs_1050°/2_. The results of the study suggested that the surface treatment of titanium disks with GNPs represents a promising solution to improve the antibacterial activity of titanium implants.

## 1. Introduction

Dental implants are one of the most commonly used ways to replace missing teeth worldwide, with high long-term success and survival rates. Nevertheless, since these devices are in close contact with hard and soft surrounding tissues, infection can occur, leading to biological complications early on during the osseointegration process, or later by inducing peri-implant diseases, such as mucositis or peri-implantitis [1,2,3]. Peri-implant diseases are an emerging problem in contemporary dentistry, with a weighted mean subject-based prevalence of 46.83% and 19.83% for mucositis and peri-implantitis, respectively [4]. Peri-implant mucositis has been defined as a reversible inflammatory reaction in the soft tissues surrounding a functioning implant, whereas peri-implantitis has been described as an irreversible inflammatory reaction associated with loss of supporting bone around an implant in function [5]. The Sixth European Workshop on Periodontology confirmed that ‘‘peri-implant diseases are infectious in nature”, and that the accumulation of microbial biofilms in dental implants plays a major role in the initiation of peri-implant diseases [6].

Different protocols have been proposed in the literature to reduce the bacterial load and remove the biofilm from the surfaces of infected implants, including mechanical, chemical, photodynamic, and laser treatments. Mechanical debridement consists in removing hard- and soft-tissue deposits with curettes, ultrasonic scalers, powdered air-abrasive systems, rubber cups, titanium brushes, and abrasive pumice [7,8,9,10]. Chemical decontamination is performed by means of topical applications of saline solution, delmopinol, chlorhexidine, cetylpyridinium chloride (CPC), tetracycline, minocycline, citric acid at pH 1, hydrogen peroxide, EDTA, or 35% phosphoric acid gel [7,8,9,10]. Photodynamic therapy (PDT) uses a photosensitizer solution in combination with diode laser irradiation to produce highly reactive singulet oxygen which can destroy bacterial cells [7,8,9,11], and laser decontamination has bactericidal effects through the denaturation of proteins by thermal effects [7,8,9,10,12]. However, none of these methods have been proven to be effective in achieving satisfactory decontamination of the implant surface, and there is no consensus about the most advantageous treatments in recovering peri-implant health [8,13]. The adjunctive use of systemic antibiotic therapy has been shown to have little or no impact on treatment success [14]. In addition, the widespread use of antibiotics may lead to adverse events and even select antibiotic-resistant bacteria [15]. Finally, it is worthy to note that pathological conditions which develop in the peri-implant tissues not only put implants and reconstructions at risk, but also may potentially affect the patient’s health [16]. Since there is no effective therapeutic approach which provides the ultimate solution to peri-implantitis or avoids the progression or recurrence of the disease, and ultimately implant loss, the only winning strategy is to prevent infection of the peri-implant tissues [17].

For the aforementioned reasons, over the last few years several surface treatments have been developed to improve the antibacterial activity of titanium implants, including nanotechnologies with antimicrobial properties, such as graphene [18,19,20,21,22,23]. 

Graphene is an atomically thick sheet composed of sp2-hybridized carbon atoms arranged in a 2D, flat hexagonal structure. Several nanomaterials which are different in terms of surface properties, number of layers, and size derive from graphene. They include few-layered graphene, ultrathin graphite, graphene oxide, reduced graphene oxide, and graphene nanosheets [24]. 

The antimicrobial effects of GNPs are due to their direct interaction with bacteria cells [25]. The mechanisms involved in the bactericidal activity are: penetrating and disrupting the cell membrane, which is due to the nano-knife action of the nanostructure’s sharp edges [26,27]; phospholipid extraction from the lipid layers of the broken bacterial membranes, which is due to Van Der Walls forces and hydrophobic properties [28]; and mechanical stress and prevention of nutrient uptake, which is related to the 2D nanostructures’ ability to wrap the cells [29]. Moreover, GNPs possess the typical anti-adhesion property of graphene, because the absence of basal plane functional groups inhibits the cell adhesion over substrate, and there is also a lower potential for cytotoxicity than graphene oxide, because the absence of oxygen-containing functional groups on the basal plane does not generate reactive oxygen species (ROS) which produce oxidative stress [30,31]. However, regarding the biocompatibility and cytotoxicity of graphene-related materials, conflicting results have been reported in the literature because the response of living cells to these nanomaterials depends greatly on their layer number, lateral size, purity, dose, surface chemistry, and hydrophilicity [32,33,34]. Nevertheless, in a previous study, Zanni et al. [35] evaluated the nanotoxicology of GNPs using the model system *Caenorhabditis elegans*, which is an excellent indicator of nanotoxicology that has highlighted no toxic impact on this animal, nor on its vitality and reproduction capability.

The aim of the present in vitro pilot study was to compare the antimicrobial activity against *Staphylococcus aureus (S. aureus)* of titanium surfaces coated with different types of GNPs colloidal suspensions in order to test the feasibility of wider experimentation for selecting the more effective antibacterial coating produced from graphite intercalated compounds, setting the temperature and duration of the thermal shock and varying the number of exfoliation cycles.

The null hypothesis is that there is no difference in antimicrobial activity against *S. aureus* of titanium surfaces coated with different types of GNPs colloidal suspensions compared to uncoated titanium disk specimens.

## 2. Materials and Methods 

### 2.1. Synthesis of GNPs and Preparation of GNPs Suspensions

GNPs were produced as described in previous papers [36,37]. Briefly, graphite intercalated compound (GIC) was used as a precursor to form worm-like expanded graphite (WEG) through a thermal driven expansion in a muffle furnace. Following the thermal treatment, two different types of WEG were produced, setting the temperature and duration of the thermal shock at 1150 °C for 5 s and at 1050 °C for 30 s, respectively. Each of the two types of WEG were dispersed in ethanol and exfoliated through a different number of tip sonication cycles by using an ultrasonic processor (Vibracell VCX750, Sonics and Materials, inc., Newtown, CT, USA) operating at 20 kHz for 20 min, with ultrasound amplitude set at 70% and operating at 15 °C in pulsed mode (1 s off, 1 s on).

Six different colloidal suspensions of GNPs in ethanol were obtained:

1. GNPs from 1 exfoliation cycle of WEG expanded at 1150 °C for 5 s (GNPs _1150°/1_).
2. GNPs from 2 consecutive exfoliation cycles of WEG expanded at 1150 °C for 5 s (GNPs_1150°/2_)
3. GNPs from 3 consecutive exfoliation cycles of WEG expanded at 1150 °C for 5 s (GNPs_1150°/3_)
4. GNPs from 1 exfoliation cycle of WEG expanded at 1050 °C for 30 s (GNPs_1050°/1_)
5. GNPs from 2 consecutive exfoliation cycles of WEG expanded at 1050 °C for 30 s (GNPs_1050°/2_)
6. GNPs from 3 exfoliation cycles of WEG expanded at 1050 °C for 30 s (GNPs_1050°/3_) (Table 1).

### 2.2. Experimental Specimens’ Preparation

Titanium disks (Ti-disks), 10 mm × 5 mm in thickness with the sand-blasted and acid-etched surface (Micro Rough Surface^®^ WinSix^®^, BioSAFin S.r.l., Ancona, Italy), were sprayed using an airbrush with 2 mL of each of six different colloidal GNPs suspensions, then air-dried and sterilized by UV rays. The UV ray method was selected for its sporicidal and virucidal activity when used for surface sterilization in the absence of organic matter, such as blood and saliva [38,39]. A total of 60 specimens, 10 for each type of suspension, were prepared. Ti-disks were provided in a sterile package by BioSAFin S.r.l., Ancona, Italy).

### 2.3. Field Emission-Scanning Electron Microscope Analysis

All Ti-disk surfaces were characterized through a Field Emission-Scanning Electron Microscope (FE-SEM) using an Auriga FE-SEM (Zeiss, Oberkochen, Germany), available at the Sapienza Nanotechnology and Nanoscience Laboratory. The FE-SEM images were used to evaluate the size (µm^2^) of the GNPs and the percentage of Ti-disks surface-coated by the GNPs. Image J (Java-based image processing program) was used for image analysis. To determine the reproducibility of the spraying process, the GPN coating of Ti-disk surfaces was carried out at two different times. For each suspension of colloidal GNPs, the mean surface coating percentage of the first five Ti-disks was compared to the mean percentage of the second group of five Ti-disks. 

### 2.4. Antibacterial Assay 

The antibacterial activity of the specimens was assessed against Gram-positive *Staphylococcus aureus ATCC 25923*, as previously described by Zanni et al. [40]. The biofilm growth in 12-well microtiter plates was estimated by using the crystal violet (CV) assay, a dye specific to biofilm biomass. In each well, which contained one Ti-disk coated by a different type of GNP, 2 mL of a suspension containing *S. aureus* cells (with a final concentration 1 × 10^7^ cell/mL) and Tryptic Soy Broth medium (TSB, Becton–Dickinson and Company, Franklin Lakes, NJ, USA) with 2% glucose (to stimulate biofilm formation) was pipetted. After incubation of the plates at 37° C for 24 h, the culture medium was removed, and the wells with Ti -disks were washed twice with H_2_O_2_ to remove the non-adherent bacteria. The plates were then kept at 65 °C for 20 min. Finally, the Ti-disks were stained with 0.3% Crystal Violet (Sigma-Aldrich) and incubated at room temperature for 15 min. After several washes with H_2_O_2_, plates were left to dry and the Ti-disks were then treated with 2 mL of 96% EtOH for CV elution. Absorbance at 560 nm was then measured by using a multiplate reader (GloMax^®^multi+detection system, Promega Corporation, Madison, WI 53711 USA). 

### 2.5. Statistical Analysis

Data were evaluated using standard statistical analysis software (version 20.0, Statistical Package for the Social Sciences, IBM Corporation, Armonk, NY, USA). A database was created using Excel (Microsoft, Redmond, WA, USA). Descriptive statistics including mean ± SD values were calculated for each variable. The Shapiro–Wilk test was used to determine whether or not the data conformed to a normal distribution. The Independent Samples T-Test was used to determine the reproducibility of the spraying process. The one-way repeated-measures ANOVA was used to identify statistically significant differences in the size (µm^2^) of the GNPs, in the percentage of Ti-disk surfaces coated by the GNPs, and in the biofilm formation on the coated Ti-disks. Pairwise comparisons were performed with Tukey correction for multiple comparisons. In each test, the cut-off for statistical significance was *p* ≤ 0.05.

## 3. Results

### 3.1. Morphological Characterization of GNPs

**Figure 1:**
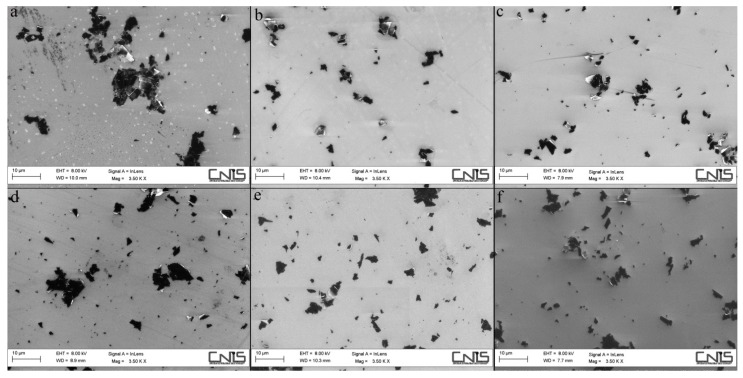
FE-SEM images of the size (µm2) of the GNPs in the six different groups: (**a**) GNPs_1050°/1_, (**b**) GNPs_1050°/2_, (**c**) GNPs_1050°/3_, (**d**) GNPs_1150°/1_, (**e**) GNPs_1150°/2_, (**f**) GNPs_1150°/3._

**Figure 2:**
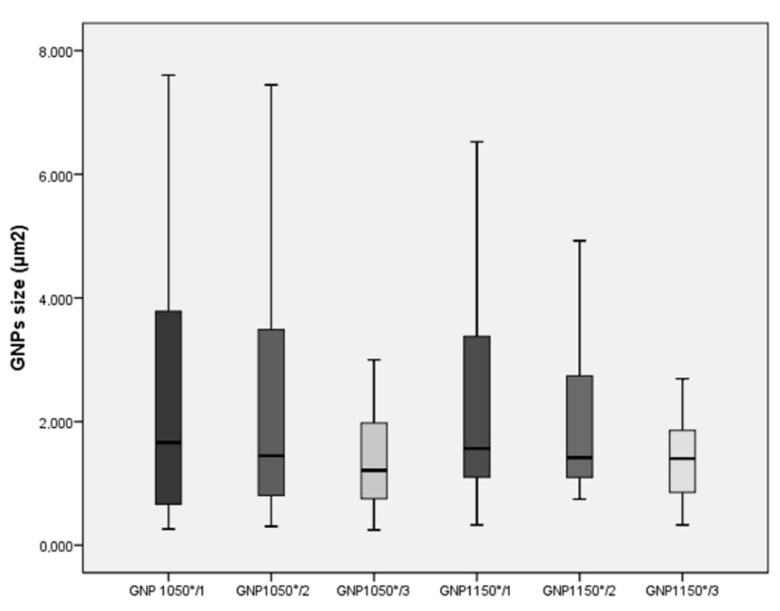
Box plots showing the median, quartile, and minimum and maximum values of the size (µm^2^) of the GNPs in the six different groups. Boxes contain 50% of all values; the horizontal lines inside the boxes indicate the medians and the vertical lines extend to 1.5 of the interquartile range.

The six different GNPs were evaluated using extensive FE-SEM investigations providing the mean size of flakes and percentage of Ti-disk surfaces coated by the GNPs. The dimension of GNPs decreased progressively after each sonication cycle from 2.21 ± 1.50 µm^2^ to 1.40 ± 0.67 µm^2^ in the GNPs_1150°_ groups, and from 2.54 ± 2.35 µm^2^ to 1.36 ± 0.73 µm^2^ in the GNPs_1050°_ groups (Figure 1 and Figure 2). Statistically significant differences in size were found between GNPs_1050°/1_ and GNPs_1050°/3_ (*p* = 0.008), and between GNPs_1050°/1_ and GNPs_1150°/3_ (*p* = 0.012) (Table 2). 

### 3.2. Morphological Characterization of Ti-Disk Surfaces Coated by GNPs

**Figure 3:**
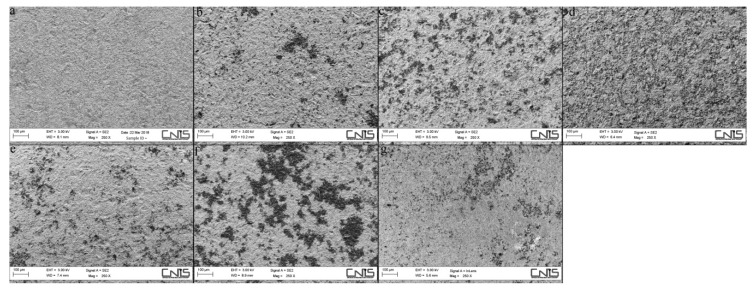
FE-SEM images of Ti-disk surfaces coated by the six different types of GNPs: (**a**) untreated Ti-disk surface (**b**) GNPs _1050°/1_, (**c**) GNPs_1050/2_, (**d**) GNPs_1050°/3_, (**e**) GNPs_1150°/1,_ (**f**) GNPs_1150°/2_, and (**g**) GNPs_1150°/3_.

**Figure 4:**
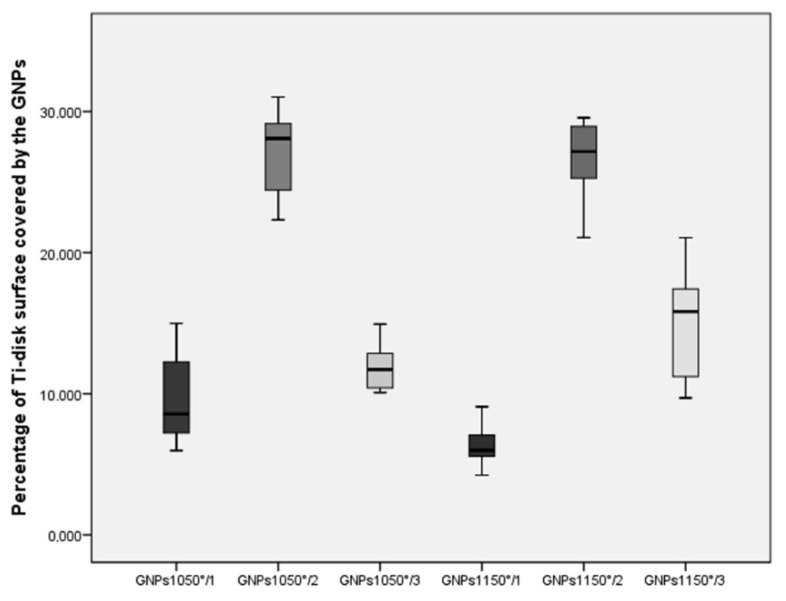
Box plots showing the median, quartile, and minimum and maximum values of the percentage of Ti-disk surfaces coated by the six different types of GNPs. Boxes contain 50% of all values; the horizontal lines inside the boxes indicate the medians, and the vertical lines extend to 1.5 of the interquartile range.

No statistically significant difference in the mean surface coating percentage of Ti-disks was found within individual groups, attesting to the reproducibility of the spraying process. The mean percentage of Ti-disk surfaces coated by the GNPs were 6.47 ± 1.55% in the GNPs_1150°/1_, 26.66 ± 2.8% in the GNPs_1150°/2_, 14.99 ± 3.90% in the GNPs_1150°/3_, 9.50 ± 3.10% in the GNPs_1050°/1_, 27.25 ± 2.79% in the GNPs_1050°/2_, and 14.99 ± 1.23% in the GNPs_1150°/3_ (Figure 3 and Figure 4), respectively. The statistically significant difference in the percentage of Ti-disk surfaces coated by the GNPs is reported in Table 3. 

### 3.3. Antimicrobial Activity

**Figure 5:**
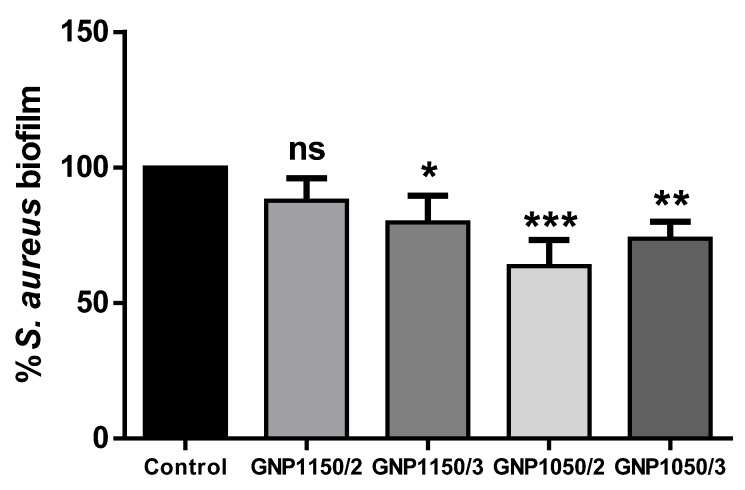
Biofilm formation was analyzed by the crystal violet binding assay in *S. aureus* cells. The production of bacterial biomass was evaluated. The production of bacterial biomass was evaluated for each of the four groups of Ti-disks coated by GNPs, and expressed as biofilm formation relative to uncoated Ti-disk samples (control group). Statistical significance: * *p* < 0.05, ** *p* < 0.01, *** *p* < 0.001, and *ns* = not significant.

Based on the previous results on morphological characterization of Ti-disk surfaces coated by GNPs, the antimicrobial activity was investigated only in Ti-disks coated by GNPs_1150/2_, GNPs_1150°/3_, GNPs_1050°/2_, and GNPs_1050°/3_, excluding GNPs_1150°/1_ and GNPs_1050°/1_ due to the poor surface coating (mean percentage < 10%). The biofilm inhibitory activity against *S. aureus* cells was evaluated after the Ti-disks coated by GNPs were incubated at 37° C for 24 h. The reduction of biofilm development compared to uncoated Ti-disks specimens was 14.4% in GNPs_1150°/2_, 20.1% in GNPs_1150°/3_, 39.2% in GNPs_1050°/2_, and 30.3% in GNPs_1050°/3_. Evidence is sufficient to reject the null hypothesis (Figure 5). 

## 4. Discussion

Over the last decade, surface modifications and coatings of titanium implants, including graphene-coated titanium surfaces, have been extensively studied in order to minimize bacterial adhesion, inhibit biofilm formation, and provide effective killing of bacteria [41,42]. The numerous papers published in the literature differ in opinion, including the present study, in terms of graphene derivatives, transferring techniques, textures of the titanium surface, tested microbial species, incubation conditions for biofilm growth, and methods for quantifying the amount of biofilm. This makes difficult to compare current results with those reported in previous papers, although some generalizations can be made. The GNP production process represents a viable solution for large-scale development of anti-biofilm devices, being low-cost, highly scalable, non-toxic, and non-oxidizing [36,37], when compared to a dry transfer technique based on a hot-pressing method [20], chemical vapour deposition coupled with a wet technique transfer (using polymethyl methacrylate or PMMA) [43], electroplating and ultraviolet reduction methods [44], evaporation-assisted electrostatic assembly processes and a mussel-inspired one-pot assembly process [45], and cathodal electrophoretic deposition [46]. Furthermore, differences in antibacterial properties might depend on the specimens (Ti-disks with commercially available sand-blasted and acid-etched surfaces) and microbial species (*S. aureus*) used in the present study, which were different from commercial pure Ti-plates or solid titanium and microbial species, such as *Streptococcus mutans, Enterococcus faecalis, Porphyromanas gengivalis,* and *Escherichia coli,* which have been used in other studies.

The preliminary results supported the hypothesis that the surface treatment of Ti-disks with GNPs may represent a promising solution to improve the antibacterial activity of titanium dental implants, due to the mechanical interaction with the bacterial cell membrane of the sharp edges of the nanostructured graphene protruding from the coating, and to the characteristic biofilm anti-adhesion effect of graphene [30,37]. In order to select the method for achieving the more effective antibacterial coating of titanium dental implants, six different colloidal suspensions of GNPs were produced, setting two different temperatures and durations of the thermal shock and six different sonication cycles of the exfoliation. The temperature and time duration of the expansion phase and the number of sonication cycles of the exfoliation greatly affects the morphological characteristic of GNPs [47], and the flake dimensions can largely influence the antimicrobial activity of the GNPs [37].

In the present study, the dimension of GNPs decreased progressively after each sonication cycle in both groups expanded at 1150 °C for 5 s and at 1050 °C for 30 s, with a statistically significant difference found between GNPs_1050°/1_ and GNPs_1050°/3_ (*p* = 0.008) and between GNPs_1050°/1_ and GNPs_1150°/3_ (*p* = 0.012). In GNPs produced with the same methodology, Rago et al. [37] obtained flakes with a smaller size (average lateral dimension 2–3 μm) when GIC was expanded at 1050 °C for 30 s, compared to flakes (mean lateral size around 5–6 μm) obtained at a higher temperature and shorter time (1150 °C for 5 s). This difference was justified by the lower expansion temperature and longer exposition time allowing for the prevention of sp2 bond degradation of the graphene lattice, and simultaneously, a more extensive reaction of the intercalating groups [37]. 

When morphological characterization of Ti-disk surfaces coated by GNPs was considered, the mean percentages of the surface coating was low in all specimens. This low percentage of GNP coating might have been due to the sand-blasted and acid-etched surface of Ti-disks both for the roughness, which could have prevented an effective graphene transfer, and for chemical changes, which could have affected the adhesion energy between the graphene and the titanium surface [48].

The best results, which were found in GNPs_1150°/2_ (26.66 ± 2.8%) and in GNPs_1050°/2_ (27.25 ± 2.79%) demonstrated that the performance of GNPs in surface coating depended more on the number of exfoliation sonication cycles (two consecutive exfoliation cycles) than on the temperature and time duration of the expansion phase (WEG expanded respectively at 1150°C for 5 s and at 1050 °C for 30 s).

To investigate the antimicrobial activity of Ti-disks coated by GNPs, *S. aureus* as a microbial model and Crystal Violet (CV) staining to quantify the amount of biofilm formed on the material surfaces were chosen. *S. aureus* is one of the most common pathogens implicated in implant infection and may be of importance in the development of peri-implantitis induced by bacterial infection [48]. *S. aureus* is known to have the ability to attach to almost any type of titanium surface, and it has been found more frequently in peri-implantitis sites than in healthy implants with a range of 0–43.4% vs. 0 to 19.1%, respectively [49,50,51]. The two main methods reported in the literature to quantify the amount of biofilm are Confocal Laser Scanning Microscopy (CLSM) and Crystal Violet (CV) staining. The first is an optical imaging technique used to obtain high-resolution images of biofilms at various depths within a sample as well as to generate 3D reconstructions of the sample, which is especially useful for fully hydrated, living specimens [52,53]. Crystal Violet (CV) staining, which has been widely applied in the literature, is a quick and reliable screening method used to examine the impact of numerous compounds on cell survival and growth inhibition [45,54].

In antibacterial evaluation, two groups of specimens (GNPs_1150°/1_ and GNPs_1050°/1_) which were produced with only one exfoliation cycle of WEG and that expanded at 1150°C for 5 s and at 1050 °C for 30 s, were excluded, because the mean percentage of the surface coating was <10%. All the other groups showed a reduction in biofilm development compared to uncoated Ti-disk specimens, with the best results being found when the WEG which expanded at 1050°C for 30 s were exfoliated through two (39.2% in GNPs_1050°/2_) or three (30.3% in GNPs_1050°/3_) sonication cycles. Similar results were reported when the antibacterial activity of GNPs suspensions, obtained starting from GNPs_1150°_ and GNPs_1050°_ on a clinical isolate of *S. mutans* after 24 h of treatment were tested [37]. Reduced survival of the cells was observed for both types, although the GNP_1050°_ showed the highest reduction. A possible explanation for this is the tighter adherence to the cell surface due to the improved exfoliation of GNP_1050°_ when compared to GNP_1150°_, which enhanced the effect of cell trapping and wrapping [37]. 

In addition, the best results obtained in the present study against *S. aureus* from the GNPs_1050°/2_ and GNPs_1050°/3_ groups might be correlated not only to the smaller size of the flakes, but also to the quality and quantity of the nanostructures’ distribution onto the Ti-disk surfaces, which mechanically damaged the bacterial walls and cell membranes [26,27,28]. Therefore, repeated spraying processes of colloidal GNP suspensions on Ti-disks should increase the percentage of surface covered by GNPs, improving its antibacterial properties. Nevertheless, the GNPs_1050°/2_ group which had the best performance in the reduction of biofilm development was lower than the 56% reported by polymer dental adhesives filled by GPNs suspension produced with the same technology. The difference could be explained by the different adhesion and protrusion of the nanostructures onto the sand-blasted and acid-etched surface of Ti-disks when compared to the teeth samples, and by the tested microbial species (*Staphilococcus aureus* v/s *Streptococcus mutans*) [36].

## 5. Conclusions

The technology tested in the present in-vitro pilot study has shown that titanium surfaces coated with GNPs have antibacterial effects and can help to prevent implant infection. However, there are some limitations implied in the experimental phase which aimed to prove the feasibility of the coating process on the Ti-disks and to test antimicrobial effects only through the reduction of biofilm development. 

Further research will need to optimize the surface coating of commercially available implants by GNPs for improving antibacterial potency, to confirm long-term and broad-spectrum antimicrobial effects, to check GNPs cytotoxicity, and to investigate the adherence and differentiation of osteoblasts for demonstrating osseointegration.

## Figures and Tables

**Table 1:** Preparation of GNP suspensions.

Name of GNP Suspensions	Temperature of Expansion (°C)	Duration of Thermal Shock (s)	Number of Sonication Cycles	Duration of Each Sonication Cycle (min)
GNPs_1150°/1_	1150	5	1	20
GNPs_1150°/2_	1150	5	2	20
GNPs_1150°/3_	1150	5	3	20
GNPs_1050°/1_	1050	30	1	20
GNPs_1050°/2_	1050	30	2	20
GNPs_1050°/3_	1050	30	3	20

GNPs: graphene nanoplatelets; °C: Celsius; s: seconds; min: minutes.

**Table 2:** Differences in sizes of the six GNP groups.

Multiple Comparisons						
Dependent Variable: GNPs size (µm^2^) Tukey HSD						
(I)	(J)	Mean Difference (I−J)	Std. Error	Sig.	95% Confidence Interval	
					Lower Bound	Upper Bound
GNP_1050°/1_	GNP_1050°/2_	0.308175	0.341085	0.945	−0.67193	1.28828
	GNP_1050°/3_	1.179900 *	0.341085	0.008	0.19980	2.16000
	GNP_1150°/1_	0.324350	0.341085	0.933	−0.65575	1.30445
	GNP_1150°/2_	0.485100	0.341085	0.714	−0.49500	1.46520
	GNP_1150°/3_	1.138875 *	0.341085	0.012	0.15877	2.11898
GNP_1050°/2_	GNP_1050°/1_	−0.308175	0.341085	0.945	−1.28828	0.67193
	GNP_1050°/3_	0.871725	0.341085	0.113	−0.10838	1.85183
	GNP_1150°/1_	0.016175	0.341085	1.000	−0.96393	0.99628
	GNP_1150°/2_	0.176925	0.341085	0.995	−0.80318	1.15703
	GNP_1150°/3_	0.830700	0.341085	0.148	−0.14940	1.81080
GNP_1050°/3_	GNP_1050°/1_	−1.179900 *	0.341085	0.008	−2.16000	−0.19980
	GNP_1050°/2_	−0.871725	0.341085	0.113	−1.85183	0.10838
	GNP_1150°/1_	−0.855550	0.341085	0.126	−1.83565	0.12455
	GNP_1150°/2_	−0.694800	0.341085	0.325	−1.67490	0.28530
	GNP_1150°/3_	−0.041025	0.341085	1.000	−1.02113	0.93908
GNP_1150°/1_	GNP_1050°/1_	−0.324350	0.341085	0.933	−1.30445	0.65575
	GNP_1050°/2_	−0.016175	0.341085	1.000	−0.99628	0.96393
	GNP_1050°/3_	0.855550	0.341085	0.126	−0.12455	1.83565
	GNP_1150°/2_	0.160750	0.341085	0.997	−0.81935	1.14085
	GNP_1150°/3_	0.814525	0.341085	0.165	−0.16558	1.79463
GNP_1150°/2_	GNP_1050°/1_	−0.485100	0.341085	0.714	−1.46520	0.49500
	GNP_1050°/2_	−0.176925	0.341085	0.995	−1.15703	0.80318
	GNP_1050°/3_	0.694800	0.341085	0.325	−0.28530	1.67490
	GNP_1150°/1_	−0.160750	0.341085	0.997	−1.14085	0.81935
	GNP_1150°/3_	0.653775	0.341085	0.395	−0.32633	1.63388
GNP_1150°/3_	GNP_1050°/1_	−1.138875 *	0.341085	0.012	−2.11898	−0.15877
	GNP_1050°/2_	−0.830700	0.341085	0.148	−1.81080	0.14940
	GNP_1050°/3_	0.041025	0.341085	1.000	−0.93908	1.02113
	GNP_1150°/1_	−0.814525	0.341085	0.165	−1.79463	0.16558
	GNP_1150°/2_	−0.653775	0.341085	0.395	−1.63388	0.32633

* The mean difference is significant at the 0.05 level.

**Table 3:** Differences in the percentage of Ti-disk surfaces covered by the six types of GNPs.

Multiple Comparisons						
Dependent Variable: Percentage of Ti-disks Surface Covered by the GNPs Tukey HSD						
(I)	(J)	Mean Difference (I−J)	Std. Error	Sig.	95% Confidence Interval	
					Lower Bound	Upper Bound
GNP_1050°/1_	GNP_1050°/2_	−17.756200 *	1.228707	0.000	−2.138639	−1.412601
	GNP_1050°/3_	−2.455500	1.228707	0.357	−608.569	117.469
	GNP_1150°/1_	3.029000	1.228707	0.153	−60.119	665.919
	GNP_1150°/2_	−17.160200 *	1.228707	0.000	−2.079039	−1.353001
	GNP_1150°/3_	−5.493000 *	1.228707	0.001	−912.319	−186.281
GNP_1050°/2_	GNP_1050°/1_	17.756200 *	1.228707	0.000	1.412601	2.138639
	GNP_1050°/3_	15.300700 *	1.228707	0.000	1.167051	1.893089
	GNP_1150°/1_	20.785200 *	1.228707	0.000	1.715501	2.441539
	GNP_1150°/2_	0.596000	1.228707	0.997	−303.419	422.619
	GNP_1150°/3_	12.263200 *	1.228707	0.000	863.301	1.589339
GNP_1050°/3_	GNP_1050°/1_	2.455500	1.228707	0.357	−117.469	608.569
	GNP_1050°/2_	−15.300700 *	1.228707	0.000	−1.893089	−1.167051
	GNP_1150°/1_	5.484500 *	1.228707	0.001	185.431	911.469
	GNP_1150°/2_	−14.704700 *	1.228707	0.000	−1.833489	−1.107451
	GNP_1150°/3_	−3.037500	1.228707	0.151	−666.769	59.269
GNP_1150°/1_	GNP_1050°/1_	−3.029000	1.228707	0.153	−665.919	60.119
	GNP_1050°/2_	−20.785200 *	1.228707	0.000	−2.441539	−1.715501
	GNP_1050°/3_	−5.484500 *	1.228707	0.001	−911.469	−185.431
	GNP_1150°/2_	−20.189200 *	1.228707	0.000	−2.381939	−1.655901
	GNP_1150°/3_	−8.522000 *	1.228707	0.000	−1.215219	−489.181
GNP_1150°/2_	GNP_1050°/1_	17.160200 *	1.228707	0.000	1.353001	2.079039
	GNP_1050°/2_	−596.000	1.228707	0.997	−422.619	303.419
	GNP_1050°/3_	14.704700 *	1.228707	0.000	1.107451	1.833489
	GNP_1150°/1_	20.189200 *	1.228707	0.000	1.655901	2.381939
	GNP_1150°/3_	11.667200 *	1.228707	0.000	803.701	1.529739
GNP_1150°/3_	GNP_1050°/1_	5.493000 *	1.228707	0.001	186.281	912.319
	GNP_1050°/2_	−12.263200 *	1.228707	0.000	−1.589339	−863.301
	GNP_1050°/3_	3.037500	1.228707	0.151	−59.269	666.769
	GNP_1150°/1_	8.522000 *	1.228707	0.000	489.181	1.215219
	GNP_1150°/2_	−11.667200 *	1.228707	0.000	−1.529739	−803.701

* The mean difference is significant at the 0.05 level.
